# Effect of Dental Implant Metal Artifacts on Accuracy of Linear Measurements by Two Cone-Beam Computed Tomography Systems Before and After Crown Restoration

**Published:** 2017-11

**Authors:** Hoorieh Bashizadeh Fakhar, Roxana Rashtchian, Milad Parvin

**Affiliations:** 1 Assistant Professor, Dental Research Center, Dentistry Research Institute, Tehran University of Medical Sciences, Tehran, Iran; Department of Oral and Maxillofacial Radiology, School of Dentistry, Tehran University of Medical Sciences, Tehran, Iran; 2 Postgraduate Student, Department of Oral and Maxillofacial Radiology, School of Dentistry, Tehran University of Medical Sciences, Tehran, Iran; 3 Postgraduate Student, Department of Oral and Maxillofacial Surgery, School of Dentistry, Tehran University of Medical Sciences, Tehran, Iran

**Keywords:** Artifacts, Cone-Beam Computed Tomography, Dental Implants

## Abstract

**Objectives:**

The aim of this study was to determine the impact of fixture location and crown restoration on the accuracy of linear measurements by two cone-beam computed tomography (CBCT) systems.

**Materials and Methods:**

Six dental implants were inserted in a dry human mandible in two stages. CBCT images were obtained in each stage by Alphard VEGA 3030 and Promax 3D Max systems. Imaging procedures were repeated after metallic crown placement. Two observers measured the alveolar height and width using five radiopaque markers. Values were compared to the same measurements made on initial images (prior to implant insertion) using t-test. The linear regression test was used to evaluate the effect of implant location on the accuracy of linear measurements.

**Results:**

The impact of fixture and fixture-crown combination on the accuracy of linear measurements of height (t = −5.2, P=0.0001 and t=−5.98, P<0.0001, respectively) and width (t=−3.42, P=0.004 and t= −2.7, P=0.015, respectively) was significantly underestimated. Metal crowns had no significant effect on measurements of bone height and width (t=−1.38, P=0.19 and t=0, P=1.00, respectively). Although both systems showed some underestimations, Promax 3D Max underestimated bone width significantly more than the other system (Alphard VEGA 3030=− 0.51mm and Promax 3D Max=−0.80). Regarding implant location, the measurements in the canine sites were found to be more accurate than the region between adjacent implants.

**Conclusions:**

CBCT is an accurate and reproducible system for dental implant follow-up examinations. Metal artifacts can lead to underestimation of measurements. However, this was not statistically significant in our study.

## INTRODUCTION

Recently, cone beam computed tomography (CBCT) has become the modality of choice for pre-surgical dental implant examinations [1]. It is also used for postoperative evaluations to assess the clinical signs and symptoms of peri-implantitis and evaluate the marginal bone level and bone-implant contact [2]. However, presence of metal in the area increases the risk of artifacts, which decrease image quality. Sometimes, the artifacts render the image useless [3–5]. Exposure parameters can have a great role in producing artifacts by affecting the energy of photons; in this context, some studies have recommended imaging techniques with high voltage (kVp) to decrease beam hardening [5–7]. Other important factors are degree of rotation of the CBCT unit, configuration of X-ray beam and type of algorithms used for data processing [5].

Numerous studies have evaluated metal artifacts in computed tomography (CT) [8–10] and CBCT images [5, 11, 12]; however, majority of them have been qualitative reports. Since there are different techniques to reduce these artifacts in CBCT imaging [13–16], only few studies have investigated the effect of metal artifacts on the accuracy of quantitative alveolar bone width and height measurements adjacent to dental implants [17, 18]. The aim of the present study was to evaluate the effect of metal artifacts produced by dental implants, before and after crown restoration, on the accuracy of linear measurements using two CBCT units.

## MATERIALS AND METHODS

**Fig. 1:**
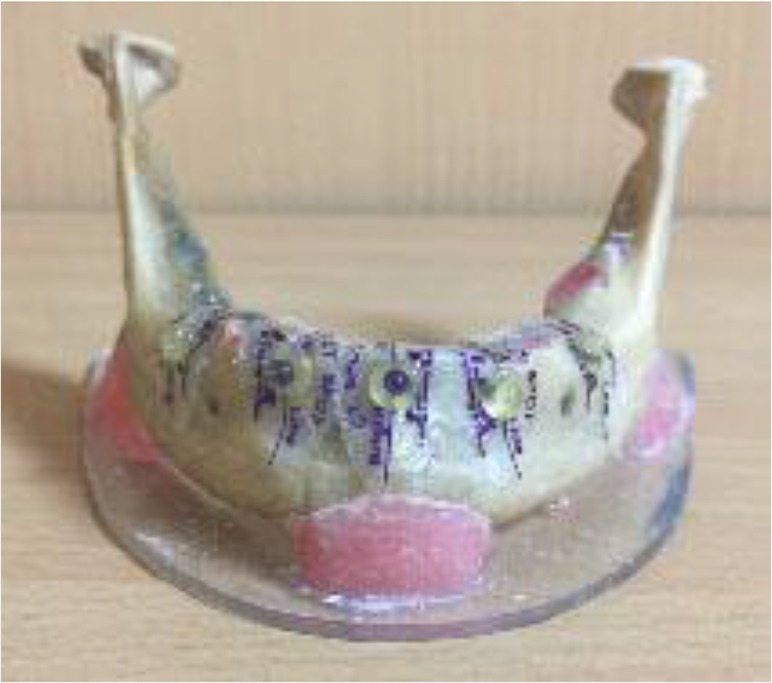
Placement of radiopaque markers in the corresponding regions in a dry human mandible

In this in-vitro study, five regions [the midline (n=1), canine sites (n=2), and first molar sites (n=2)] were marked by 3 mm radiopaque spheres attached to the superior border of the alveolar buccal bone of a dry edentulous human mandible (Fig. 1). Prior to implant insertion, the gold standard for linear measurements was provided by scans of the mandible using both CBCT units. Since alveolar width and height values were similar and no significant differences were found, the measurements were considered as the gold standard. In order to simulate soft tissue attenuation, the mandible was placed in a water-filled plastic container (diameter of 12 cm, height of 8 cm, wall thickness of 3 mm). To increase stability, the mandible was fixed to an acrylic tripod plate so that the occlusal plane could be adjusted parallel to the horizon. Tapered Implants (Super line; Dentium, Implantium, Seoul, Korea), with 8 mm height and 4 mm diameter were used to evaluate artifacts. Two implants were placed in the canine sites, two in the second premolar sites and two in the second molar sites using a surgical guide.

CBCT images were obtained using the following systems:

1: Alphard VEGA 3030 (Asahi Rontgen Ind. Co., Ltd, Kyoto, Japan), with the exposure protocol of: voxel size = 0.2 mm, 80 kVp, 5 mA, 10x10 cm field of view, and 17 sec. exposure time.
2: Promax 3D Max (Planmeca, Helsinki, Finland), with exposure protocol of: voxel size= 0.2 mm, 74 kVp, 12 mA, 10x5.5 cm field of view, and 12.26sec exposure time.

**Fig. 2:**
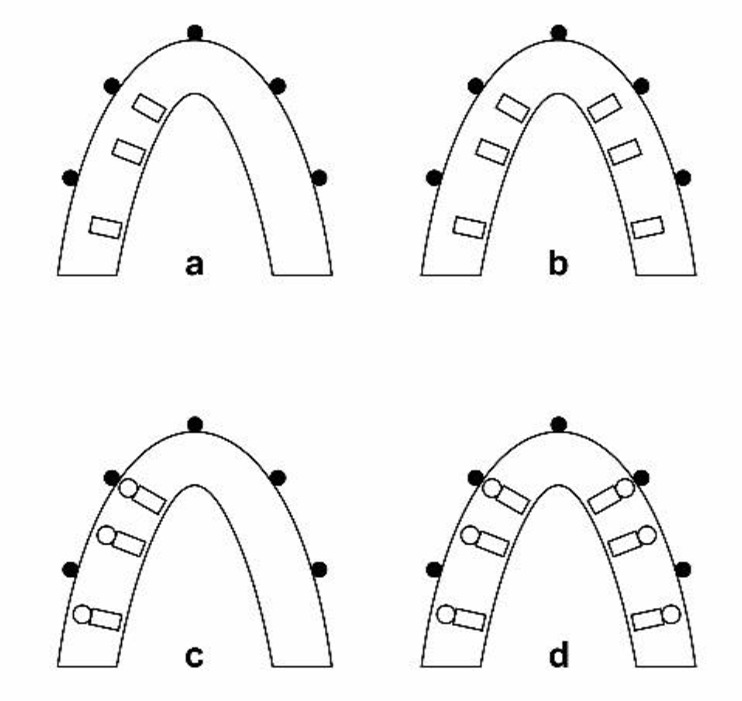
Schematic view of imaging stages used in CBCT devices from a to d (rectangular shapes, fixture; white round shapes, metal crowns; black round shapes, radiopaque markers)

The imaging process was as follows (Fig. 2):

- Three fixtures were drilled in the right side of the mandible at canine, second premolar and second molar sites.
- Additional three fixtures were drilled on the left side of the mandible at the same mentioned sites.
- Fixtures were removed from the left side and a direct impression was made from the right site. The nickel-chromium crowns were fabricated and cemented.
- Left side fixtures were inserted again; the nickel-chromium crowns were fabricated and cemented.

**Fig. 3:**
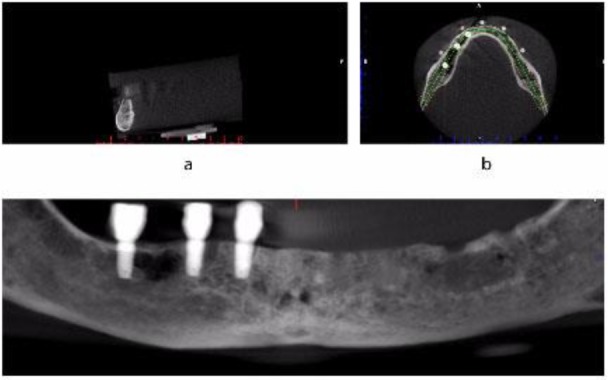
A drawn panoramic line in the software at the third imaging stage: (a) Sagittal (b) Axial (c) Panoramic-reconstructed image

Images were obtained at each stage using both CBCT units. Once the imaging process was completed, a panoramic line was manually drawn in the axial plane while passing through the center of implants, using the NEO 3D software (Asahi Rontgen Ind., Co., Ltd., Kyoto, Japan) for Alphard VEGA 3030 unit and Romexis software version 2.9.2 (Planmeca, Helsinki, Finland) for Promax 3D Max unit (Fig. 3). Then, the cross-sectional images were reconstructed through the centers of radiopaque markers, and coded according to the markers (from 1 to 5).

**Fig. 4:**
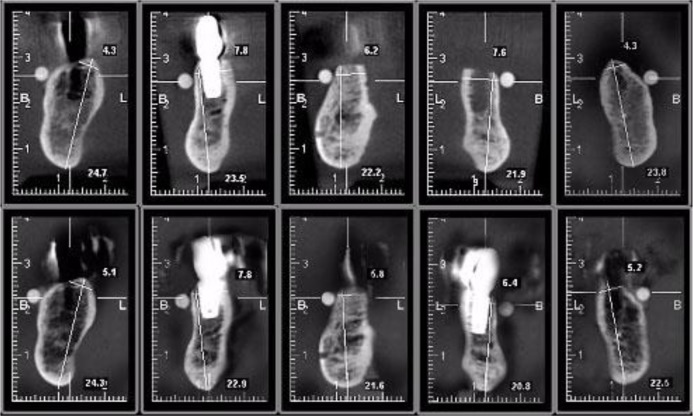
Cross-sectional images of third and fourth imaging stages from left to right corresponding to markers

In each cross-sectional image, two regions of Interest (ROIs) were identified: ROI1 was defined as the alveolar height, measuring from the buccal alveolar crest to the lowest border of the mandible; and ROI2 was defined as the alveolar width, locating 1.5 mm inferior to the tangent of buccolingual aspect of alveolar crest (Fig. 4). Images were evaluated twice with an interval of two weeks by two oral and maxillofacial radiologists, and the obtained measurements were compared with the gold standard.

### Statistical analysis:

The STATA software version 12 (STATA Corporation, College Station, Texas, USA) was used for data analysis. The Intraclass correlation coefficient (Pearson’s correlation) was used to determine the interobserver agreement and intraobserver reliability. Paired t-test was used for pairwise comparisons. The linear regression test was used to evaluate the effect of metal artifacts on the accuracy of linear measurements at different markers’ positions. The level of significance was set at P≤0.05.

## RESULTS

The Interobserver agreement was ICC=0.878 for ROI1, and 0.881 for ROI2 (P<0.001). The intraobserver agreement was ICC= 0.853 for ROI1 and 0.865 for ROI2 (P<0.001). These results were satisfactory.

### The Effect of crown restoration:

In the first imaging stage, the effect of fixtures on the accuracy of linear measurements of ROI1 and 2 at the left side (markers 4 and 5) was evaluated. The measurements were found to be underestimated (0.75, and 0.44, respectively). The differences were statistically significant (t=−5.2, P=0.0001 and t=−3.42, P=0.004, respectively).

In the third imaging stage, the effect of fixture-crown combination on the accuracy of linear measurements of ROI1 and 2 at the left side of the mandible (markers 4 and 5) was evaluated. The measurements were found to be underestimated (0.81, 0.5mm, respectively). The differences were statistically significant (t=−5.98, P<0.0001 and t=−2.7, P=0.015, respectively). According to the comparison between fixture-crown combination and fixture alone (comparison between the first and third imaging stages), the difference between measurements of ROI1 and 2 at the left side (markers 4 and 5) was not significant (t=−1.38, P=0.19 and t=0, P=1.00, respectively).

### Effect of implant location (Table 1):

Since the fixtures were placed bilaterally in the mandible (with and without crowns in the fourth and second imaging stages, respectively), linear regression model was used to compare the difference between the ROIs and the gold standard at midline, canine, and molar sites; the effect of observer, CBCT system, and presence of crown was modified.

**Table 1:** Mean difference between the measured values and the gold standard according to different markers

	**Marker**	**Mean difference (cm)**	**SD**
Width	1 (Molar area on the right side)	−0.34	0.55
	2 (Canine area on the right side)	0.19	0.74
	3 (Midline)	−1.16	0.45
	4 (Canine area on the left side)	0.00	0.44
	5 (Molar area on the left side)	−0.84	0.72

Height	1 (Molar area on the right side)	−1.03	0.4
	2 (Canine area on the right side)	−0.53	0.76
	3 (Midline)	−0.81	0.47
	4 (Canine area on the left side)	−0.41	0.39
	5 (Molar area on the left side)	−0.66	0.79

SD: Standard deviation

### Comparisons between ROI2 and gold standard in the midline and canine sites:

The difference was 1.34 mm, which was significant (P<0.05). Although the measurement was underestimated in the midline and overestimated at the canine site, the absolute error was less at the canine sites.

### Comparisons between ROI1 and gold standard in the midline and canine sites:

The difference was 0.32 mm, which was significant (P<0.05). Linear regression analysis showed that measurements at the implant sites were more accurate than those between adjacent implants in the anterior region.

### Comparisons between ROI2 and the gold standard in the midline and molar sites:

The difference was 0.72 mm, which was significant (P<0.05). The measurements were underestimated at both sites, but the absolute error was less in the molar sites.

### Comparisons between ROI1 and the gold standard in the midline and molar sites:

The difference was 0.18 mm, which was not significant (P=0.06).

### Comparisons between ROI2 and gold standard in the canine and molar sites:

The difference was 0.63 mm, which was significant (P<0.05). The absolute error was less in the canine sites.

### Comparisons between ROI1 and the gold standard in the canine and molar sites:

The difference was 0.4 mm, which was significant (P<0.05). The measurements were more accurate at the implant sites compared to those between adjacent implants in the posterior region.

### Effect of CBCT system (Table 2):

The measured values of different CBCT systems were underestimated compared to the gold standard (width and height); the differences were significant (P<0.05).

**Table 2:** Effect of different CBCT units on linear measurements

	**Mean difference of bone width with gold standard (cm)**	**(95% CI)**	**Mean difference of bone height with gold standard (cm)**	**95% CI**
**Alphard VEGA**	0.51	0.4–0.62	0.79	0.65–0.92
**Promax 3D Max**	0.80	0.66–0.94	0.70	0.57–0.85

The mean (standard deviation) values of ROI1 in different systems are shown in Table 2; there was no statistically significant difference (P=0.729). The mean (standard deviation) values of ROI2 in different systems showed that Promax 3D Max tended to underestimate the values (0.35mm) compared to Alphard VEGA system (P=0.01).

## DISCUSSION

CBCT imaging is useful in two phases of treatment: pre-surgical implant imaging (e.g. determines the quality and quantity of alveolar bone height and width, and assists in selection of dental implants) and post-surgical implant imaging [2, 19].

Metal artifacts complicate CBCT interpretation. In general, when a polychromatic X-ray beam passes through an object, low energy photons are absorbed more than high energy photons. This leads to an increase in the mean X-ray beam energy and beam hardening [12].

Since back projection algorithms are used to reconstruct three-dimensional images in CT and CBCT units, artifacts can be observed in both. Compared to MDCT, CBCT units have more artifacts with shorter streaks; this may be ascribed to the different geometry of beam radiation [12, 20].

Currently, three methods are available for evaluating the accuracy of measurements made by the use of CBCT units: geometric hardware phantom, anthropomorphic phantom, and comparison with MDCT. It was found that CBCT is highly accurate and reproducible for linear measurements [21–27]. Thus, in the current study with high reliability and agreement between the observers, we evaluated the accuracy of alveolar measurements in four imaging stages and compared them with the gold standard. To determine the effect of metal artifacts, before and after crown reconstruction, we assessed the accuracy of linear measurements in the 1^st^ and 3^rd^ imaging stages. Although metal crowns can lead to greater underestimation of measurements, but the differences were not significant.

There are two forms of beam hardening artifacts in CBCT imaging, including cupping artifact, and missing value or extinction artifact. It has been reported that implants may lead to an increase in gray values in proximity to buccal surfaces of anterior and posterior implants [6, 7]. Thus, we placed radiopaque markers on the canine sites, to assess the effect of cupping artifact on the accuracy of measurements. Previous studies concluded that the extinction artifacts were more commonly observed between adjacent implants [3, 28]. Therefore, we made the measurements in the midline and first molar sites.

Linear regression model was used to assess the accuracy of CBCT measurements in different sites. Regarding bone width, the impact of extinction artifact was more than that of cupping artifact. Since the alveolar width measurements in the canine sites were slightly overestimated due to the implant’s shadow, the absolute error was less than that between adjacent implants (in the midline and molar sites). Besides, the alveolar height measurements were found to be more accurate in the canine sites than those between adjacent implants.

In this study, we evaluated the effect of different CBCT systems on the accuracy of linear measurements. In terms of alveolar height, there was no significant difference between Alphard 3030 and Promax 3D Max systems. In terms of alveolar width, Promax 3D Max underestimated the values significantly more than Alphard 3030. Liedke et al. [29] evaluated the factors that affect the conspicuity of the buccal bone condition around dental implants in CBCT systems. They found that the implant-abutment material was the most relevant factor. Acquisition and reconstruction factors had minor impact on detection of buccal bone condition. In our study, given the similarity of other parameters (types of dental implants and crowns, voxel size and number of implants), it seemed that factors such as the number of projections (due to the lower rotation of Promax 3D Max) and lower voltage (kVp) of Promax 3D Max might be the most important factors.

Brown et al. [30] and Neves et al. [31] found that reducing the number of image projections did not affect the accuracy of CBCT measurements. However, their studies were not comparable to ours, because they did not use any metal object in their phantoms, which is a confounding factor. Esmaeili et al. [5] showed that in presence of metal artifacts, a higher image quality was observed with NewTom VG than with Planmeca CBCT system. In the current study, the effect of metal artifacts on the accuracy of CBCT images was evaluated quantitatively.

Leung et al. [32] reported that horizontal and vertical measurements of animal bones were underestimated (mean difference was 0.3mm) using Helical CT and CBCT units. Similarly, in the present study, horizontal and vertical measurements were underestimated by use of both systems. However, we showed greater mean measurement error that may be due to the presence of metal artifacts.

Razavi et al. [33] evaluated the accuracy of measuring the cortical bone thickness adjacent to dental implants using two CBCT systems (i-CAT NG, Accuitomo 3D FPD). They found that the measurements of cortical bone thickness were underestimated and the measurements of marginal level were overestimated. In the current study, we found that the alveolar width and height measurements were underestimated. It seems that methods which tend to underestimate are safer and more acceptable than those leading to overestimation of measurements [32].

Patcas et al. [18] found that CBCT was more accurate for linear measurements than MDCT. However, partial volume and beam hardening effects do not allow reliable application of CBCT solely for post-implant evaluation. Thus, at present, periapical radiography continues to be the method of choice for assessment of bone loss around dental implants in follow-up examinations; CBCT images can be requested as an adjunctive imaging in cases where signs of failure, paresthesia or infection are present.

## CONCLUSION

The accuracy of CBCT measurement is influenced by metal restorations (e.g. fixture and metal crown), implant location and the CBCT system. Metal artifacts may lead to underestimation of measurements. However, this underestimation was less than 1mm in our study, which makes them clinically acceptable.
